# Systematic identification of intergenic long-noncoding RNAs in mouse retinas using full-length isoform sequencing

**DOI:** 10.1186/s12864-019-5903-y

**Published:** 2019-07-08

**Authors:** Ying Wan, Xiaoyang Liu, Dongwang Zheng, Yuying Wang, Huan Chen, Xiaofeng Zhao, Guoqing Liang, Dongliang Yu, Lin Gan

**Affiliations:** 1 College of Life and Environmental Sciences, Hangzhou Normal University, Hangzhou, China; 2 Zhejiang Key Laboratory of Organ Development and Regeneration, Hangzhou Normal University, Hangzhou, China; 3 Zhejiang Academy of Medical Sciences, Hangzhou, China; 4 Key Laboratory of microbiological technology and Bioinformatics in Zhejiang Province, Hangzhou, China; 5 Department of Ophthalmology and Flaum Eye Institute, University of Rochester, Rochester, NY 14642 USA

## Abstract

**Background:**

A great mass of long noncoding RNAs (lncRNAs) have been identified in mouse genome and increasing evidences in the last decades have revealed their crucial roles in diverse biological processes. Nevertheless, the biological roles of lncRNAs in the mouse retina remains largely unknown due to the lack of a comprehensive annotation of lncRNAs expressed in the retina.

**Results:**

In this study, we applied the long-reads sequencing strategy to unravel the transcriptomes of developing mouse retinas and identified a total of 940 intergenic lncRNAs (lincRNAs) in embryonic and neonatal retinas, including about 13% of them were transcribed from unannotated gene loci. Subsequent analysis revealed that function of lincRNAs expressed in mouse retinas were closely related to the physiological roles of this tissue, including 90 lincRNAs that were differentially expressed after the functional loss of key regulators of retinal ganglion cell (RGC) differentiation. In situ hybridization results demonstrated the enrichment of three class IV POU-homeobox genes adjacent lincRNAs (*linc-3a*, *linc-3b* and *linc-3c*) in ganglion cell layer and indicated they were potentially RGC-specific.

**Conclusions:**

In summary, this study systematically annotated the lincRNAs expressed in embryonic and neonatal mouse retinas and implied their crucial regulatory roles in retinal development such as RGC differentiation.

**Electronic supplementary material:**

The online version of this article (10.1186/s12864-019-5903-y) contains supplementary material, which is available to authorized users.

## Background

Vertebrate retinas shares a highly organized and conserved structure, and is an excellent system to study the differentiation and development of neurons [1, 2]. Mouse (*Mus musculus*) retinas contain six major types of neurons and one type of glia that are involved in optical to visual signal transformation [3]. All retinal cells are generated from common retinal progenitor cells (RPCs) through conserved and concerted actions of a series of transcription factors, particularly components of homeodomain and bHLH families (e.g., Pax6, Six3 and Math5) [4–6]. Besides, increasing pieces of evidence in the last decade point to the crucial regulatory roles of non-coding RNAs (ncRNAs) in retinal development and diseases, such as miRNAs (e.g., *miR-124a*, with its primary sequence termed *Rncr3*) and long noncoding RNAs (lncRNAs) [7–9].

LncRNAs are defined as a class of RNAs greater than 200 nucleotides in length and without detectable coding potential [10]. LncRNAs are classified into several groups, including antisense, intronic and intergenic (lincRNAs) according to the relationship between lncRNAs and their associated protein-coding genes [11]. It is now widely accepted that lncRNAs play pivotal regulatory roles in diverse biological processes, such as imprinting, cell cycle regulation, cell differentiation, and other developmental processes [12, 13]. During mouse retinogenesis, for instance, *Tug1* is necessary for photoreceptor formation [14], *Miat* (also known as *Rncr2* or *Gomafu*) and *Six3OS* are involved in regulating the retinal cell fate specification [15, 16], *Vax2os* is involved in the control of cell cycle progression of photoreceptor progenitor cells in the ventral retina [17], and *Malat1* is functional associated with retinal microvascular dysfunction and neurodegeneration [18, 19]. Moreover, a requirement for a complex comprising of miRNA (miR-183/96/182), lncRNA (*Rncr4*) and protein (RNA helicase Ddx3x) in the organization of retinal architecture is also revealed [20]. In a more recent study, 119 lncRNAs were identified with their potential roles in regulation of photoreceptor cell differentiation [21]. All these facts revealed the importance of lncRNAs during retina formation and development, however, in view of the huge number of lncRNAs in mouse genome [22, 23], our understanding about the biological roles of lncRNAs in the differentiation and development of mouse retinal neurons remains limited, and a systematic annotation of lncRNAs expressed in the retina is still lacking.

In the present work, we carried out a long-reads sequencing of transcripts expressed in mouse retinas at developmental stages encompassing the generation of all retinal cell types and performed a genome-wide analysis of the intergenic lncRNAs (lincRNAs). About one thousand lincRNAs are identified and the subsequent function and expression analysis argue for future exploration of the roles of lncRNAs in regulating retinal development.

## Methods

### Mice

Mouse experiments in this study were performed according to protocols approved by the Ethics Committee for Experimental Animals at Hangzhou Normal University (permit number: 2018069). C57BL/6 J mice were used in this study. All the used mice were provided by the experimental animal center of Hangzhou Normal University. Mice, no more than ten, were euthanasized by carbon dioxide (20% chamber volume per minute, at least 10 min) in individually ventilated cages, followed by cervical dislocation. Retinas were dissected from embryos at embryonic days of E12.5, E14.5, E16.5 and E18.5, and from postnatal mice at P0, P3, P5, P7, P14, P21 and P28. At least three individuals were required for each developmental stage. Separation of retinas was performed according to the previously described protocol [24]. Curved scissor was used to push the eye out of its socket after killed the mouse by euthanasia. The optic nerve was cut and the eyes were removed into petri dish containing ice cold diethyl pyrocarbonate treated PBS (0.1%; Sigma, cat no: D5758). Subsequently, we used the forceps to poke a hole on the cornea and gently tear the hole to detach the retina away from the retinal pigmented epithelium. Then, the sclera, iris, lens and any other remaining structures were carefully removed until the retina was completely isolated. The isolated retinas were put into 1.5 ml RNase-free microcentrifuge tubes and stored in − 80 °C for further experiments.

### RNA preparation and sequencing

Retinas from embryonic and postnatal individual were respectively pooled prior to RNA isolation. The total RNAs were isolated using the RNeasy kits (Qiagen, Valencia, CA) in accordance with the manufacturer’s protocol, respectively. RNA degradation and contamination were monitored on 1% agarose gels. The purity of RNA samples was checked using the NanoPhotometer spectrophotometer (IMPLEN, CA, USA). RNA concentration was measured using Qubit RNA Assay Kit in Qubit 2.0 Flurometer (Life Technologies, CA, USA). The integrity of RNA was assessed using the RNA Nano 6000 Assay Kit of the Agilent Bioanalyzer 2100 system (Agilent Technologies, CA, USA). 2 μg qualified RNAs (OD260/280: 2.0–2.2, OD260/230: 1.8–2.1 and RIN ≥ 9.0) from each of the pooled embryonic and postnatal mouse retinas were mixed.

A total of 4 μg mixed RNA was used for full-length transcript sequencing with Pacbio Sequel system (Pacific Biosciences, CA, USA) according to the manufacturer’s instructions. The Iso-Seq library was prepared according to the Isoform Sequencing (Iso-Seq) protocol using the Clontech SMARTer PCR cDNA Synthesis Kit and the BluePippin Size Selection System as described by Pacific Biosciences (PN 100–092–800-03). Another 1.5 μg mixed RNA was used for short reads sequencing on HiSeq X Ten platform (Illumina, CA, USA), with the library constructed using NEBNext UltraTM RNA Library Prep Kit for Illumina (NEB, USA) following manufacturer’s recommendations.

### Iso-seq data processing and error correction

Raw data produced by Pacbio was processed by the SMRT Link package (v5.0) (http://www.pacb.com/support/software-downloads/). Reads with a length < 200 nt or with the ReadScore < 0.75 were filtered out before generating the circular consensus sequence (CSS) (minPasses = 1, minPredictedAccuracy = 0.8). FLNC (full-length non chimera) and NFL (non full-length) reads were then classified by checking the signals of 5′- and 3′- primers, as well as the poly-A tail. ICE algorithm was used to cluster the FLNC reads to generate the consensus sequences, which were then polished using the NFL reads. LoRDEC (Version 0.6) was used to correct these consensus sequences with the Hiseq generated short reads [25], which were priory filtered to remove the reads with the ambiguous bases > 10% or with the low quality bases (Q-value < 20) > 50% in length.

### Identification of lncRNAs

The reference mouse genome was downloaded from GENCODE (mm10, www.gencodegenes.org). The corrected consensus sequences were mapped to the mouse genome by using GMAP (Version 2017-01-14) (−-expand-offsets 1 -B 5 -K 50000 -f samse -n 0 -t 4) [26]. Redundant sequences were removed by using Cupcake ToFU package (https://github.com/Magdoll/cDNA_Cupcake) and CD-HIT package (CD-HIT-EST, −c 0.95 -aS 0.95) [27]. The collapsed Pacbio transcripts were then mapped back to mouse genome again using STAR [28].

CPAT (v1.2.4) was used to remove the protein-coding transcripts first, i.e., any transcript with the coding probability > 0.44 was considered as a coding sequence [29]. Thereafter, open reading frames (ORFs) of the retained transcripts were detected by TransDecoder (v5.2.0). Their deduced peptides (> 100 amino acids) were compared to the known mouse proteins using BLASTP [30], which were meanwhile scanned for potential domains by using HMMER (Version 3.2.1, −-cut_ga) (hmmer.org) and PFAM domain profiles [31]. Transcripts were filtered out if their encoded peptides showed domain architectures or shared > 85% identity to known proteins with the alignment region longer than 50 amino acids. Subsequently, BLASTN was used to align the retained Pacbio transcripts to all the mRNA of characterized mouse protein-coding genes. Transcripts were regarded as partial protein-coding transcripts if > 95% identity was observed across the aligned region that covered > 50% of the query transcripts in length.

In addition, based on the bam file from STAR alignment, MatchAnnot (https://github.com/TomSkelly/MatchAnnot) was used to compare the structures between the Pacbio transcripts and the known genes. Pacbio transcripts were removed from the collection of lncRNA candidate if they were perfectly matched the protein-coding or institute RNA genes in structure (score 5). Moreover, the Pacbio transcripts exactly matched one or more introns of protein-coding genes were also filtered out. The lincRNAs were then picked out by comparing the genomic location to known genes by cuffcompare (Cufflinks package, v2.2.1) [32]. Cuffcompare was also used to compare the locations and structures of lincRNAs identified in this work to previous annotation [21, 33, 34]. Notably, for lncRNAs deposited in NONCODE, their expression in particular tissues were accepted only when the FPKM (Fragments Per Kilobase of transcript per Million fragments mapped) > 0.01. Unless otherwise specified, default parameters were selected for the bioinformatics tools mentioned above.

### Short reads mapping and abundance calculation

Short reads generated from HiSeq were mapped to the all the mappable full-length transcripts acquired in this work, as well as all the protein-coding transcripts that annotated in GENCODE, by using BOWTIE (version 1.1.2) with no more than two mismatchs allowed [35]. Abundance of lncRNAs was measured by RPKM (Reads Per Kilobase per Million mapped reads) that was calculated by in-house developed perl scripts. Significance of abundance variation between different groups was calculated using Student’s t-test function of R package (version 3.5.0).

### Function analysis of lincRNA nearby genes

According to the genome annotation information from GENCODE release, protein-coding genes within 100 kb of lincRNA loci were collected as potential *cis*-targets [36, 37]. Functional enrichment analysis was performed through the Enrichr webserver [38], with the redundant GO terms were removed by Revigo (allowed similarity 0.7) [39]. Significance of the domain enrichment was assessed by the *p*-value generated from Fisher exact test (cutoff 0.05).

### Identification of differentially expressed lincRNAs

RNA-seq data of retinas from wild type, *Math5*-null, *Isl1*-null and *Pou4f2*-null mice at E14.5 were downloaded from NCBI Sequence Read Archive (SRA), which were deposited under the accession number SRP037539 [40]. Expression level of identified lincRNAs were calculated with the same strategy as described in ‘Short reads mapping and abundance calculation’. Differentially expressed isoforms were identified by using R package DEGSeq [41], with the cutoff fold change ≥2 and p-value ≤0.05.

### Reverse transcription PCR

Reverse transcription (RT)-PCR was used to validate the transcription of novel lincRNAs. Total RNA was extracted from pooled mouse retinas the same way as described in the RNA preparation for sequencing. DNA-free RNA was reverse transcribed to cDNAs using PrimescriptTM RT reagent Kit (Takara, Code No. RR047A). All PCRs were performed in 20 μl volume containing 4 μl 5X PrimeSTAR buffer, 1.6 μl dNTP mixture (2.5 mM), 0.5 μl primers (10 μmol/L), 0.2 μl DNA polymerase (2.5 U/μl) (R010A, Takara), 1 μl cDNA and 12.2 μl nuclease-free water. The PCR products were sequenced by ABI 3730XL DNA Analyzer (ABI/Life Technologies, USA) and aligned to the mouse genome using BLAT through the UCSC Genome Browser web service (genome.ucsc.edu).

### Fluorescence in situ hybridization (FISH)

The lncRNA probe templates, with the T3 and T7 adaptors, were firstly synthesized and inserted in pUC57 vector (Genewiz, Suzhou, China) and then amplified with the M13 primers by PrimeSTAR HS DNA Polymerase (Takara, Code No. R010A) (see Additional file 8: File S1 for probe sequences). Sense and antisense of probes were transcribed with T7 and T3 RNA polymerases, respectively. DIG RNA labeling mix (Roche, 1,277,073) was used to label the probes. Probes of *Pou4f1*, *Pou4f2* and *Pou4f3* were used as the positive control, which were generated according to the method described previously [42].

Retinas from mice at E15.5 were used for detection of gene expression. The embryos at E15.5 were fixed in 4% paraformaldehyde in PBS at 4 °C for 6 h and dehydrated in 30% sucrose in PBS at 4 °C. After dehydration, the embryos were embedded in OCT (ThermoFisher Thermo NEG50, cat. no. 6506) at − 80 °C. FISH on sectioned tissues were carried out according to the previously described method [43]. Probes were detected by using Anti-digoxigenin-HRP (1:1000) (Perkin Elmer, cat. no. NEF832001EA) and Tyramide Signal Amplification system (Perkin Elmer, TSA Plus, cat. no. NEL744001KT) according to the manufacturer’s recommendation.

## Results

### Iso-seq and error correction

Sequencing of pooled mouse retinal RNAs generated a total of 5,084,169 subreads from six SMAT cells of combined SMRTbell libraries, which formed 372,267 higher quality circular consensus sequences (CCSs). 295,179 FLNC sequences, i.e., CCSs containing the 5′- and 3′- primers and polyA tails, were subsequently clustered and polished by using NFL sequences that yielded a total of 172,333 consensus sequences. LoRDEC was used to improve the quality of the Iso-seq acquired consensus sequences, using about 195 M high-quality short reads generated by RNA-seq of the pooled retinas. After error correction, the total nucleotide of the 172,333 consensus sequences was increased from 501.8 to 505.1 M bp, whilst the average length and N50 were also slightly increased (Additional file 1: Table S1).

### Identification of lncRNA genes

Based on the mapping of the corrected consensus sequences to the mouse genome by using GMAP, a total of 120,297 transcripts (≥ 200 nt) were reconstructed using the Cupcake ToFU package. CD-HIT-EST was used to filter out the transcripts varied only in the 5′ or 3′-end and generated 71,251 collapsed transcripts. 97.5% of these transcripts (69,495) were mapped to the mouse genome with STAR (error rate per base 0.01%) and were selected for further identification of noncoding transcripts.

**Fig. 1:**
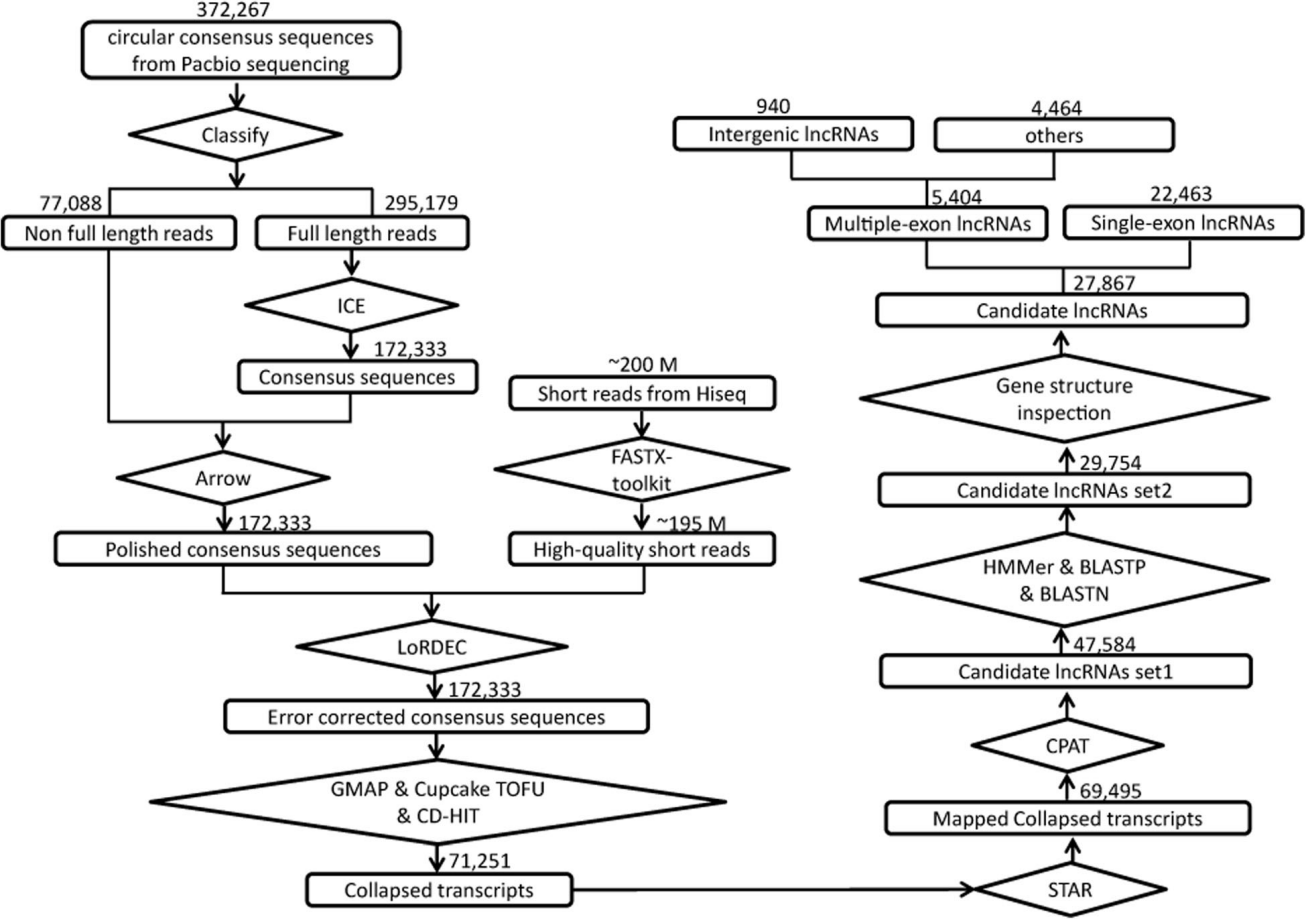
Flowchart of lincRNA identification. Full-length transcripts are generated by Pacbio Sequel system. Short reads generated from Illumina platforms are used for read quality promotion. All the full-length transcripts are retained if they could be mapped to the reference genome and their coding potential are then calculated by independent algorithms. Multiple-exon encoded lincRNAs are eventually collected for further analysis

Identification of lncRNAs in this work was performed according the strategy described in Fig. 1. First, coding potential of the candidates was calculated by CPAT, which filtered out 21,911 transcripts that showed a coding probability > 0.44. Secondly, a total of 56,126 long ORFs (with the deduced peptides > 100 amino acids) were detected in 29,027 of the remaining transcripts, including 13,523 ORFs showed significant domain features and 16,251 shared high similarity to known mouse proteins, respectively. Transcripts contained any of these ORFs were filtered out, resulting in a collection of 33,060 transcripts. Primary nucleotide sequence based homology search (BLASTN) revealed that 3306 transcripts were mapped to the characterized 5′- or 3′- untranslated regions (UTRs), which were therefore considered as fragments of protein-coding transcripts.

Moreover, exon-intron structures of the retained 29,754 transcripts were compared to annotated mouse genes with MatchAnnot. Transcripts were filtered out if their structures perfectly matched the known protein-coding / institute RNA genes (e.g., microRNAs, snoRNAs and snRNAs) or they exactly shared one or more introns with protein-coding genes, which eventually resulted in 27,867 candidate lncRNAs, including a total of 5404 ones with multiple exons. At least two short reads from the RNA sequencing of pooled retina sample were mapped to 93.4% of these candidates, i.e., 96.4% (5209/5404) and 93% (20,866/22,463) for multiple-exon and single-exon encoded candidates, respectively, indicating they were generated at low risk of DNA contamination.

Notably, transcripts derived from Iso-seq have not been subsequently mapped to specific DNA strands in this work, thus it is difficult to accurately distinguish the antisense/intronic lncRNAs from the isoforms of protein-coding genes in many cases, although a number of candidate lncRNAs could be classified into antisense/intronic lncRNAs with high confidence based on the similarity of primary sequences and structures. By checking the chromosomal locations of the identified 5404 multiple-exon encoded lncRNA candidates, 940 transcripts that non-overlapped with any known protein-coding genes were selected for subsequent analysis (Additional file 9: File S2). Exon-intron structures of ten randomly selected lincRNAs, at least one for each, were validated by RT-PCR and products analysis (Additional file 2: Table S2, Additional file 6: Figure S1).

### General features of retinal lincRNAs

**Fig. 2:**
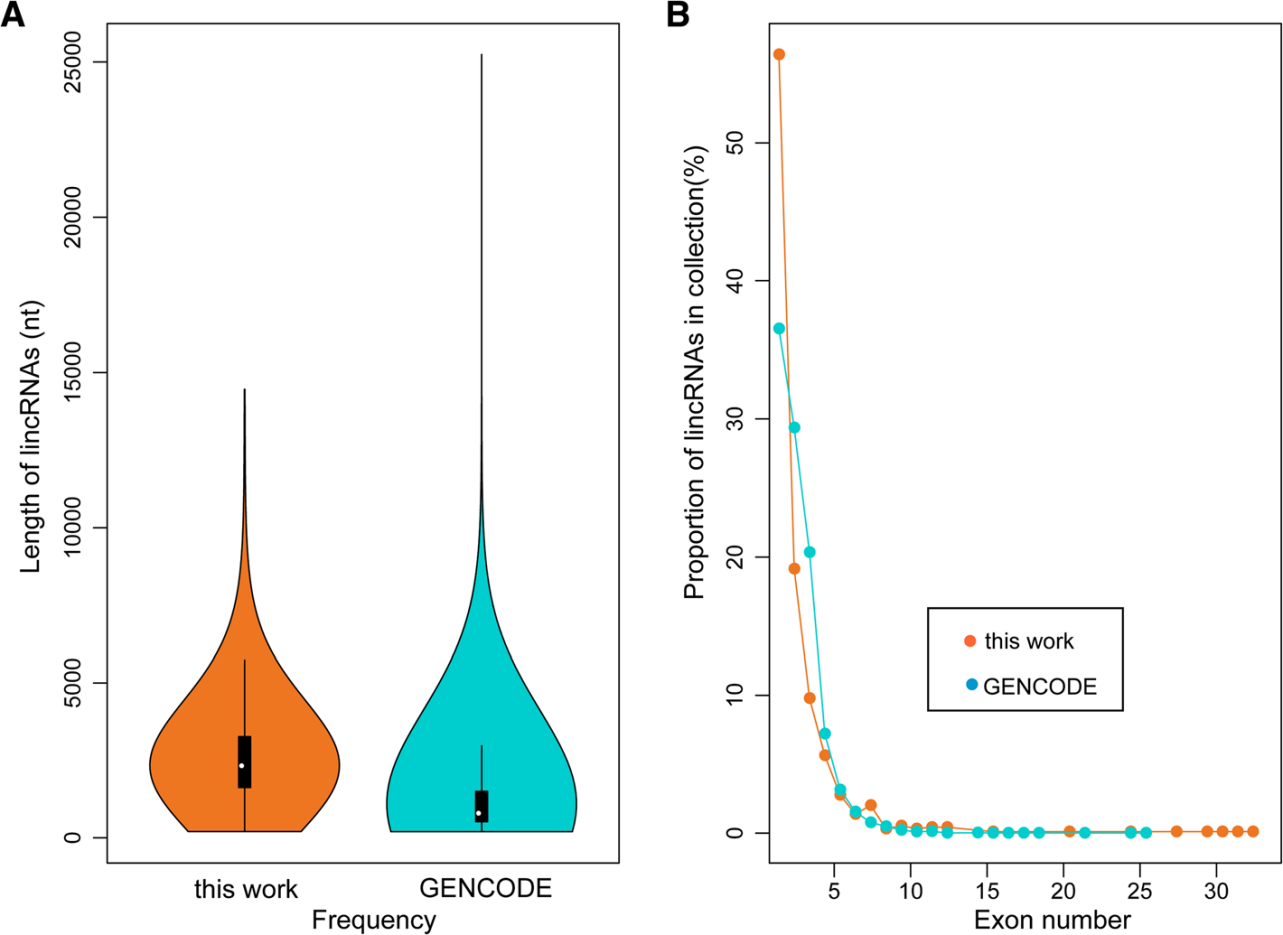
Genomic features of retinal lincRNAs. (**a**) Length distribution of lincRNAs identified in the mouse retina and annotated by GENCODE. (**b**) Statistics of exon numbers in lincRNAs identified in the mouse retina and previous annotation

The identified 940 multiple-exon encoded retinal lincRNAs were transcribed from 690 putative gene loci and their sizes ranged from 202 to 14,455 nt. Their average length of about 2600 nt was significantly longer than the known lincRNAs (Fig. 2a). Despite of the variations in length, the number of exons per lincRNA was similar between lincRNAs identified in this work and those deposited in GENCODE (vM17), i.e., about half of the lincRNAs contained two exons and > 90% lincRNAs contained fewer than five exons (Fig. 2b). Retinal lincRNAs containing the largest number of exons were transcribed from the same locus, i.e., a total of 14 lincRNAs were transcribed from a 265 kb genomic region (chr7:61,944,240- 62,209,515), including eight of them had more than ten exons.

**Fig. 3:**
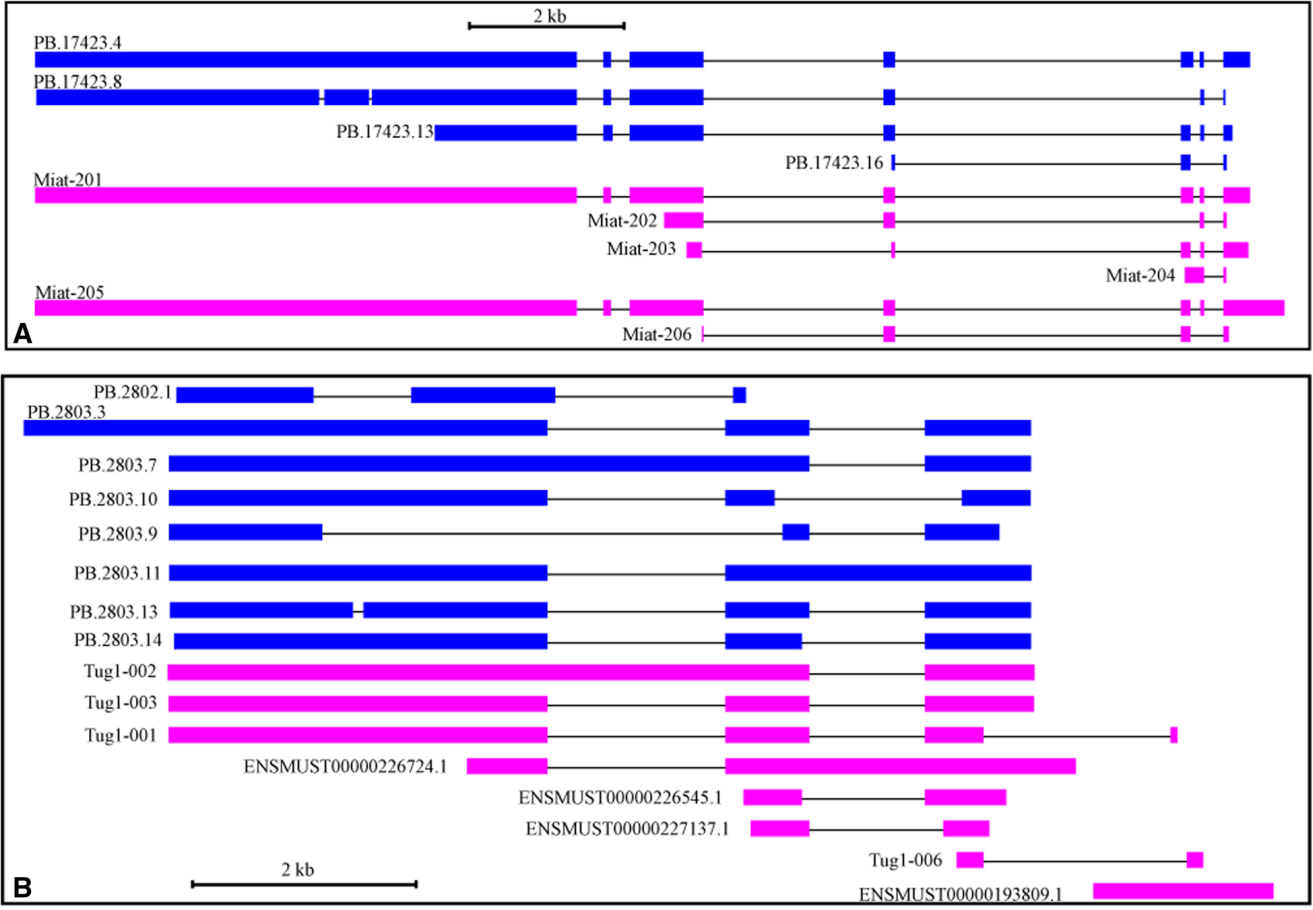
Structures of lincRNA genes *Miat* and *Tug1*. The blue and pink blocks indicate the exons of transcripts generated from Iso-seq (PB.x.x) and annotated in GENCODE (vM17), respectively. The lines between blocks represent introns. Both the exons and introns are drawn to scale. (**a**) *Miat locus;* (**b**)*Tug1 locus*

By comparing the 940 retinal lincRNAs to the GENCODE annotation, we found that about half of them (434/940) were transcribed from novel gene loci. The rest were overlapping with known lincRNAs, including 56 showed exactly the same structures to their counterparts. Taking the previously characterized lincRNA genes *Miat* and *Tug1* for example, we also observed several novel isoforms in addition to the known transcripts (Fig. 3).

The expression of mouse lncRNAs has been investigated in several tissues like brain, liver, heart, and testes, and the expression profiles could be retrieved from NONCODE database and the work of Zhao et al [33, 34]. Comparative analysis revealed that a total of 122 lincRNAs (13%), transcribed from 112 potential gene loci, were only found in mouse retinas. The other lincRNAs either shared the same structure (8.3%) or overlapped with the known lncRNAs (78.7%). In addition, we also checked the expression of those 940 lincRNAs in developmental photoreceptors (P2–28) [21]. By comparing the chromosomal location to the assembled transcripts in photoreceptors, we found that 548 (58.3%) of the identified lincRNAs were transcribed from the transcription-active loci in photoreceptors, including 75 lincRNAs that shared the same structures with the transcripts expressed in photoreceptors (Additional file 3: Table S3). In contrast, the other 392 (41.7%) lincRNAs were not overlapping with any gene expressed in photoreceptors, indicating they might be expressed in photoreceptors at the stages other than P2-P28 or in the other cell types in retina.

### Abundance of lincRNAs

**Fig. 4:**
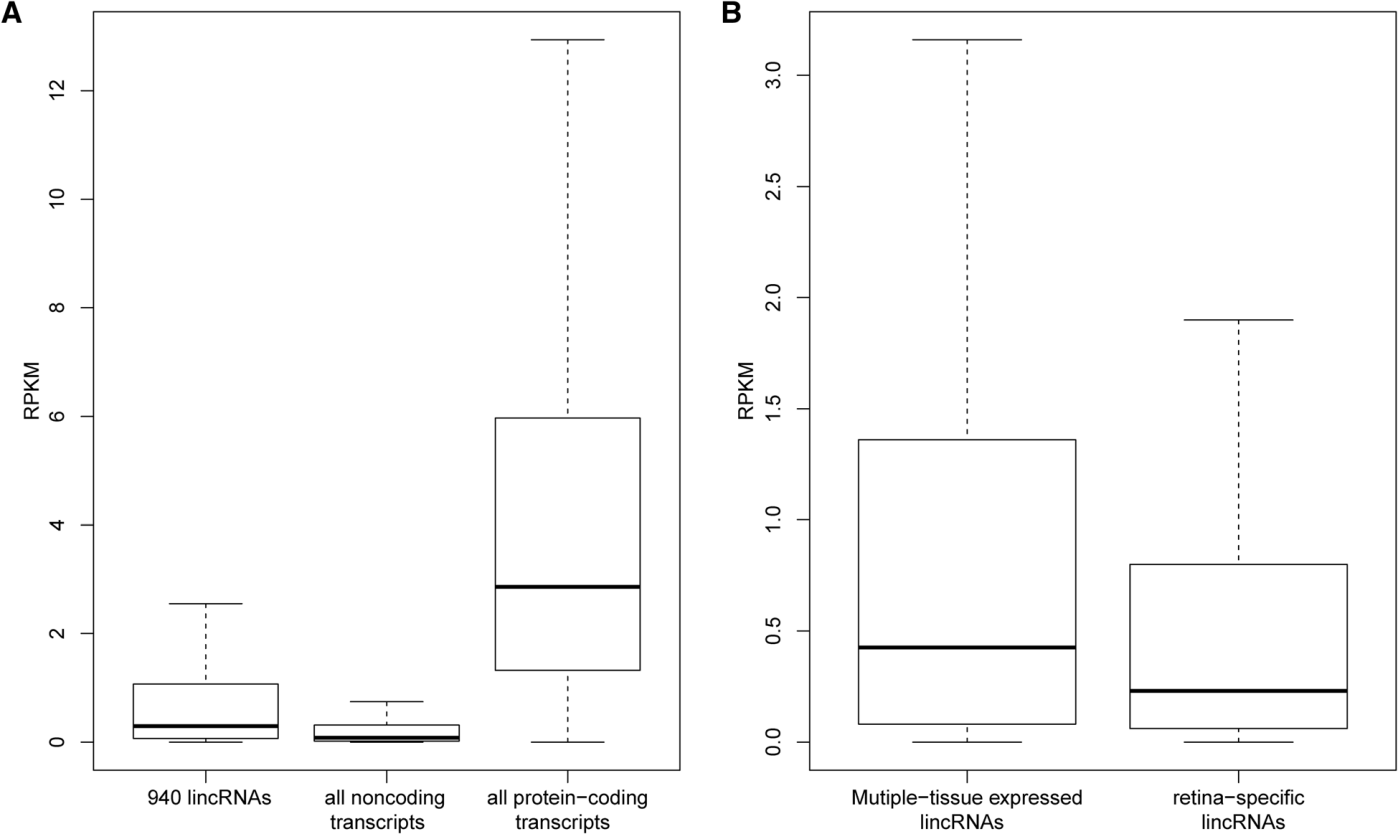
Abundance of the lincRNAs expressed in mouse retina. (**a**) Comparison of the abundance between coding/noncoding transcripts and lincRNAs. (**b**) Comparison of abundance between retina-specific and multiple-tissue expressed lincRNAs. Transcript abundance is calculated by RPKM

Short reads generated from sequencing of the pooled retinal RNAs were used to calculate the relative abundance of the lincRNAs. About 71.4% of the short reads could be aligned to 98% of the clustered full-length transcripts (69,495 in total). Consistent with the known knowledge that the noncoding RNA genes are usually expressed at a low level, the abundance of the 940 lincRNAs were significantly lower than those with coding potential, including only about 25% of them with the RPKM > 1 (Fig. 4a). Moreover, the 122 potential retina-specific lincRNAs were expressed at a remarkable lower level than multiple-tissue expressed lincRNAs (two folds in RPKM, *p* < 0.001) (Fig. 4b).

In contrary to the low expression level of most lincRNAs, several lincRNAs (non-repeat elements) were abundant in the investigated sample with the RPKM > 10 (Table 1). Interestingly, about half of these abundant lincRNAs were transcribed from *Miat*, *Malat1,* and *Rncr3*,which were involved in different processes of retinal development as mentioned above. Besides, we noticed that *Cdkn1b*, neighboring to lincRNA gene *Lockd* at ~ 3.5 kb distance, is involved in controlling the timing of cell cycle exit of retinal progenitors and is required to maintain the quiescence of bipolar cells, Müller cells, and cones [44]. Given that *cis*-regulation is a common mechanism of lincRNAs acting [45], the role of *Lockd* in retinal development is worthy of further exploration. Moreover, according to the expression profiles from NONCODE, these abundant lincRNA genes were also active in other tissues, such as liver, thymus, lung, spleen, heart or hippocampus. Notably, a half of their transcripts identified in the retina were novel that further study is required to confirm their retina-specific expression.

**Table 1:** Top 20 abundant lincRNAs expressed in the mouse retina

Transcripts	Host lncRNA gene	Relative abundance (RPKM)	Compare to GENCODE annotation
PB.20890.2	H19	83.81	known
PB.17423.4	Miat	72.23	known
PB.11213.21	Malat1	70.83	novel isoform
PB.17423.8	Miat	65.29	novel isoform
PB.17423.13	Miat	40.82	known
PB.7082.15	Rncr3	21.84	novel isoform
PB.7083.1	Rncr3	20.3	novel isoform
PB.13759.2	Mir124-2hg	20.08	novel isoform
PB.19312.10	Lockd	19.78	known
PB.5352.9	Rian	19.32	known
PB.6163.26	C130071C03Rik	18.9	known
PB.7082.1	Rncr3	18.38	known
PB.18345.2	1810058I24Rik	18.02	known
PB.12880.18	1700020I14Rik	16.48	known
PB.7082.17	Rncr3	16.43	novel isoform
PB.7082.5	Rncr3	14.23	novel isoform
PB.12880.30	1700020I14Rik	13.84	novel isoform
PB.13343.15	2900097C17Rik	12.94	novel isoform
PB.1224.18	Gas5	12.92	novel isoform
PB.13344.4	2900097C17Rik	12.74	novel isoform

### Function analysis of lincRNA neighboring genes

**Fig. 5:**
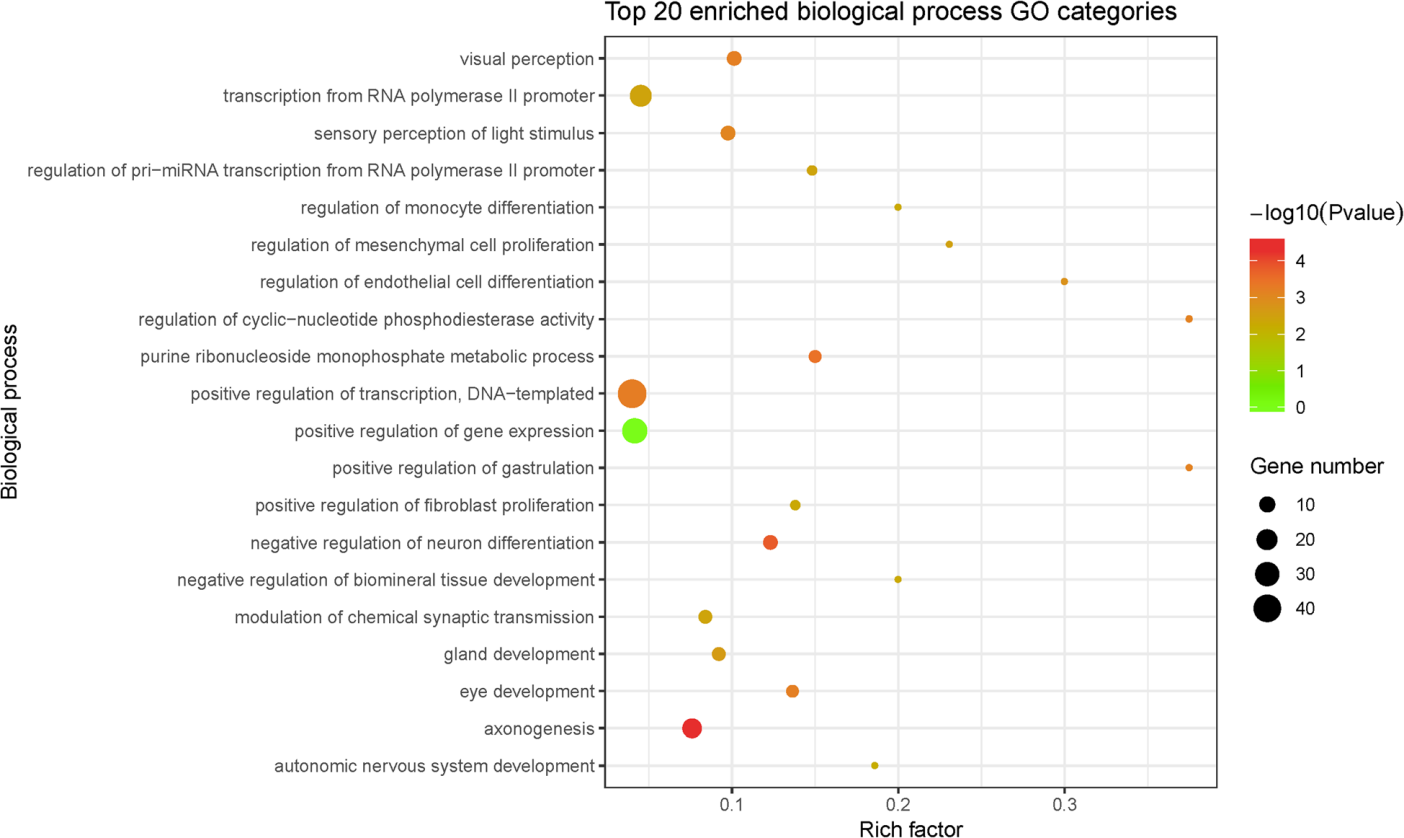
GO enrichment analysis of the retinal lincRNA-nearby genes. Nearby genes were collected within 100 kb up- or downstream of lincRNAs. Rich factors of GO terms were calculated by (gene number in the collected set) / (gene number in the whole gene set)

As high-throughput methodology directly towards the function of lincRNAs remains unavailable, biological roles of lncRNAs are usually inferred from the nearby protein-coding genes (*cis*-targets). Herein, 482 protein-coding genes nearby the identified lincRNAs were identified (within 100 kb). Results from homology search (BLASTP, ≥ 85% identity) and short reads mapping (≥2 reads) revealed that almost all of these 482 genes were expressed in developmental retinas (465/482). These potential *cis*-targets included several transcription factors that play crucial roles in retinal development, with the largest family as homeobox genes, such as *Six6*, *Lhx1*, *Onecut1*, *Six3*, *Otx2*, *Pax6*, *Pou4f1*, *Pou4f2* and *Pou4f3*. Interestingly, in this study, no lincRNA was identified nearby bHLH genes that are characterized as important regulators of retinal cell differentiation, such as *Math5*, *NeuroD*, *Mash1,* and *Hes1*. GO enrichment analysis revealed that these lincRNA neighboring protein-coding genes were largely associated with neuronal differentiation and development, e.g., the most significantly enriched biological process GO categories were axonogenesis (GO:0007409) and negative regulation of neuron differentiation (GO:0045665) (Fig. 5). Likewise, for those potentially retina specific lincRNAs, their nearby protein-coding genes were enriched in the same or related processes, such as axonogenesis, visual perception (GO:0007601) and sensory perception of light stimulus (GO:0050953) (Additional file 4: Table S4).

### Identification of lincRNAs associated with RGC regulators

MATH5, ISL1 and POU4F2 are key regulatory factors of RGCs specification and differentiation. MATH5 is required for establishing RGC competence within a subset of RPCs [46, 47]. Likewise, functional loss of *Isl1* and *Pou4f2* leads to the apoptosis of most RGCs before birth [48–51]. In addition, many other transcription factors involved in the regulation of RGC differentiation have been identified as well, such as SOX4 and SOX11 [52]. Nevertheless, little is known about the biological roles of lncRNAs during RGC differentiation and development.

In order to investigate lincRNAs that may be involved in the regulation of RGC differentiation, we analyzed the RNA-seq data of mice null for *Math5*, *Isl1* or *Pou4f2*, and found that a total of 90 lincRNAs, transcribed from 85 non-overlapped genomic regions, were differentially expressed (Table 2). Functional analysis of their nearby protein-coding genes revealed enrichment of several biological processes, such as negative regulation of neuron differentiation, axonogenesis and embryonic camera-type eye morphogenesis (Additional file 5: Table S5).

**Table 2:** A list of lincRNAs differentially expressed after the functional loss of *Math5*, *Isl1* or *Pou4f2*

Transcript	Host gene	Expression Variation (log2Foldchange)			Nearby protein-coding gene
		Pou4f2-null	Isl1-null	Math5-null	
PB.9897.1	–	−3.6	−3.6	–	1600014C23Rik
PB.1163.1	RP24-231D14.1 / Gm5532	1.6	–	–	Acbd6
PB.21043.1	–	− 1.8	− 2.8	–	Adprhl1
PB.15635.1	RP23-301 M3.1	−1.0	–	–	Ak4
PB.19692.1	–	–	− 1.1	–	Akt2
PB.15291.1	–	3.6	–	–	Aldh1b1
PB.1920.1	–	1.5	1.4	–	Atg5
PB.13742.1	RP23-3D20.2	−2.2	−4.8	−1.2	Car2
PB.235.1	–	–	–	− 1.2	Ccdc115
PB.3841.6	RP24-402O2.2	–	–	1.5	Ccdc182
PB.12472.1	–	2.6	–	2.7	Cdca7
PB.19030.1	RP23-305C1.1	–	1.1	1.0	Cntn4
PB.12202.1	–	–	1.8	–	Cntrl
PB.16460.1	–	–	1.3	1.3	Cyp51
PB.16304.1	–	2.3	–	–	Disp3
PB.2627.1	RP24-570 M12.3	−3.8	−3.8	–	Dyrk2
PB.7138.1	RP24-67I21.3	2.1	2.1	–	Entpd4
PB.13067.1	RP23-401 J24.3	–	–	−1.4	Erv3
PB.23709.1	–	–	–	− 1.5	Etd
PB.11813.1	E330013P04Rik	1.2	–	2.4	Fam204a
PB.5217.1	–	1.1	–	–	Foxn3
PB.12325.1	–	–	−1.0	–	Galnt13
PB.12831.1	RP23-230H3.7	−2.6	−2.6	–	Gjd2
PB.12831.2	RP23-230H3.2	–	3.0	–	Gjd2
PB.24411.4	–	–	–	1.6	Gm21860
PB.421.1	RP24-146 K19.1	−2.9	–	−1.5	Hsfy2
PB.11544.1	RP23-167 K23.1	–	–	2.1	Htr7
PB.5127.1	–	–	–	−2.1	Irf2bpl
PB.16019.1	–	−3.3	−3.3	–	Matn1
PB.14015.1	RP23-403H2.2	2.6	–	2.7	Mgarp
PB.12783.1	RP23-300H23.1	−1.2	–	–	Mpped2
PB.13695.1	RP24-558H21.4	–	–	−1.2	Mrps28
PB.18509.1	RP24-83C9.4	–	–	1.1	Nfe2l3
PB.22226.1	–	–	4.0	–	Nrp1
PB.17616.4	AC121564.2	–	–	1.1	Orai1
PB.7358.1	RP23-67A17.1	–	–	−3.8	Pou4f1
PB.7356.2	RP23-67A17.1	−3.0	–	− 3.6	Pou4f1
PB.7360.1	RP23-67A17.1	−2.2	–	−2.2	Pou4f1
PB.21631.1	–	–	–	−1.5	Pou4f2
PB.10720.8	–	− 2.3	–	−5.6	Pou4f3
PB.10720.6	–	−3.4	–	−5.4	Pou4f3
PB.9822.74	–	–	–	−1.0	Prrc2a
PB.4404.1	–	−1.0	−3.6	−2.0	Rab10
PB.12105.1	–	–	–	3.8	Rapgef1
PB.17233.2	–	–	–	−1.3	Rasgef1b
PB.14276.3	RP23-168E14.7	–	–	2.4	Rhbg
PB.14275.1	RP23-168E14.7	–	–	6.0	Rhbg
PB.14276.1	RP23-168E14.7	–	–	7.7	Rhbg
PB.5261.4	–	1.0	–	–	Rin3
PB.14453.1	AC131746.2	−3.9	−1.1	−1.6	Sec22b
PB.20752.2	RP23-145H5.1	−1.2	–	–	Sec23ip
PB.20752.3	RP23-145H5.1	−1.6	–	–	Sec23ip
PB.10765.1	–	–	–	−3.3	Sema6a
PB.10303.1	–	1.2	1.5	–	Six3
PB.13384.1	RP23-392P11.4	1.1	–	–	Slc32a1
PB.13385.1	RP23-392P11.4	2.6	–	–	Slc32a1
PB.18900.1	–	−1.2	–	–	Slc6a6
PB.11179.1	–	–	1.5	–	Ssh3
PB.20143.3	RP24-78 M9.4	–	–	1.3	Synm
PB.2545.1	–	1.2	–	–	Syt1
PB.20893.1	AC012382.2	4.2	–	–	Th
PB.20294.2	RP23-124O24.4	–	–	−1.2	Tmem126b
PB.580.1	–	−1.5	–	–	Tnp1
PB.2344.1	RP24-538A12.2	−1.8	–	−3.2	–
PB.11002.1	–	1.2	–	−2.8	–
PB.5665.1	RP23-235 J20.5	–	–	−2.8	–
PB.15109.1	–	− 1.2	–	− 2.6	–
PB.19035.1	–	–	–	− 2.5	–
PB.20042.1	Snhg14	–	–	−2.4	–
PB.23395.1	RP23-357C16.1	–	–	− 1.5	–
PB.10822.2	–	–	− 1.8	− 1.4	–
PB.7625.1	RP24-112I4.1	−1.6	–	−1.4	–
PB.14876.1	RP23-473G18.1	−1.8	–	− 1.1	–
PB.20300.1	RP24-267C3.1	–	–	−1.1	–
PB.6399.1	RP23-110 M15.1	–	–	− 1.0	–
PB.6163.26	RP23-88A13.2	–	–	1.1	–
PB.22575.1	RP23-442 M18.1	–	–	1.2	–
PB.1089.1	–	–	–	1.5	–
PB.23389.1	–	–	–	2.0	–
PB.313.1	RP23-176 M9.1	–	–	2.1	–
PB.13925.1	–	− 3.3	–	2.4	–
PB.8561.1	RP23-127A21.3	5.1	–	4.0	–
PB.17246.1	–	–	1.4	–	–
PB.2422.6	Rmst	−1.0	–	–	–
PB.2474.1	RP24-447 J10.1	2.2	–	–	–
PB.4459.2	RP23-329 M17.1	−4.8	−4.8	–	–
PB.4689.1	–	–	1.5	–	–
PB.6169.1	–	− 1.5	–	–	–
PB.6894.35	RP23-268P13.4	–	− 1.5	–	–
PB.7962.1	–	–	3.6	–	–

**Fig. 6:**
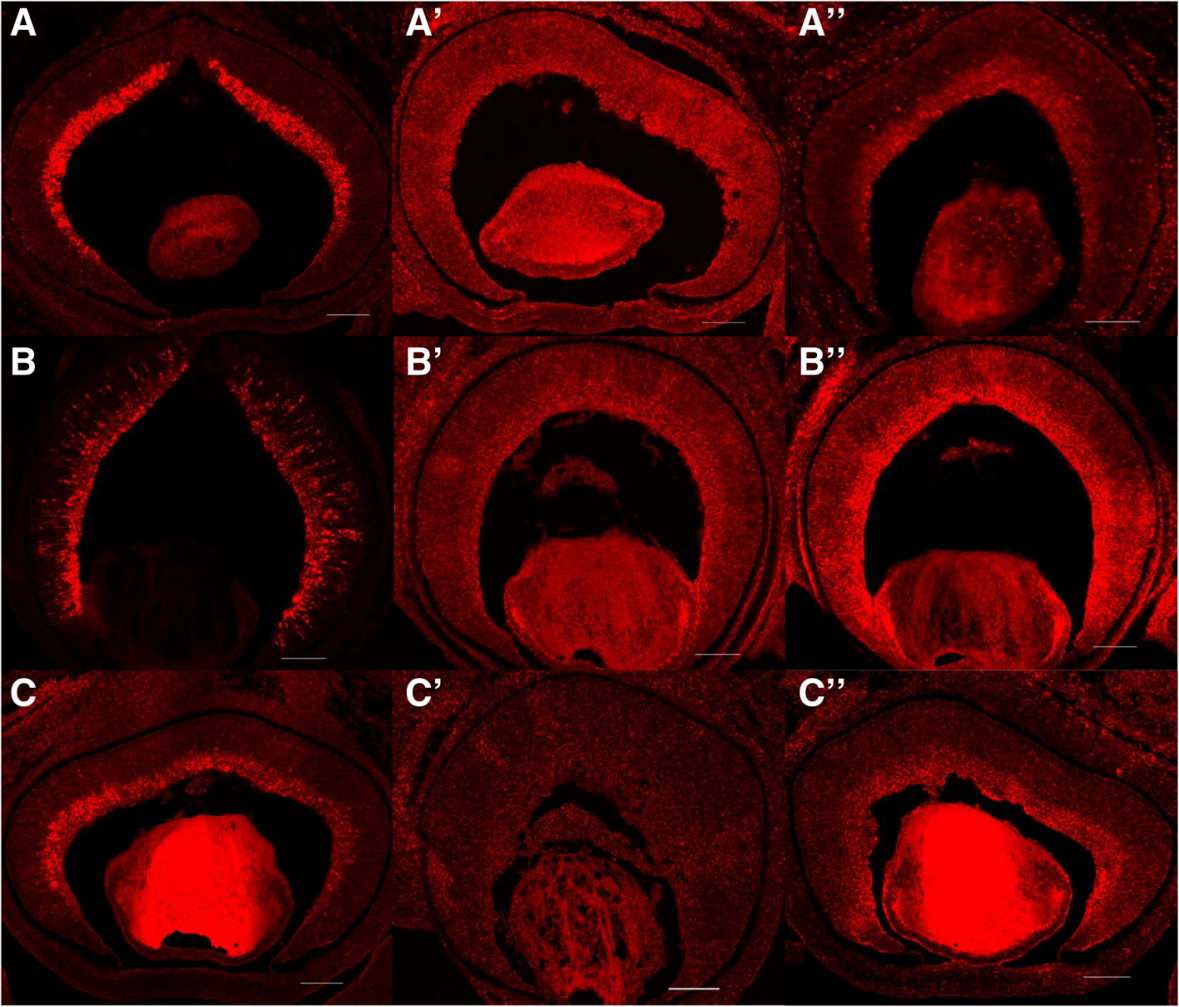
Expression of lincRNA *linc-3a*, *linc-3b* and *linc-3c* in mouse retinas at E15.5. Expression of *Pou4f1*, *Pou4f2* and *Pou4f3* were detected in the ganglion cell layer (**a**-**c**). Negative controls were performed using the probes of sense sequences of *linc-3a*, *linc-3b* and *linc-3c*, respectively (A’-C′). Expression of *linc-3a*, *linc-3b* and *linc-3c* were enriched in the ganglion cell layer (A”-C″). Scale bars: 100 μm

In *Math5*-null retinas, 22 and 31 lincRNAs were up- and down-regulated, respectively. As the targeted deletion of *Math5* leads to the agenesis of almost all RGCs, it is possible that the down-regulation of some lincRNAs were caused by the loss of RGCs. Expression of two thirds of these down-regulated lincRNAs (21/31) were not detected in photoreceptors, including three of them, i.e., PB.7356.2 (*linc-3a*), PB.21631.1 (*linc-3b*), and PB.10720.6 (*linc-3b*), were resided next to the RGC specific expressed genes *Pou4f1*, *Pou4f2* and *Pou4f3* (within 10 kb), respectively [53]. Different versions of mouse genome annotation have specified the lncRNA gene *D130079A08Rik* / *RP23-67A17.1* that overlapped *linc-3a*, as well as *Gm31501* / *Gm31438* that overlapped *linc-3c* (Additional file 7: Figure S2), whereas *linc-3b* was identified in this work. Interestingly, by using FISH detection in retinal sections, our further work demonstrated that expression of *linc-3a, linc-3b and linc-3b* were enriched in the ganglion cell layer in mouse retina that, together with the above facts, strongly indicated their RGC specific expression (Fig. 6).

Similar analysis also revealed that the expression of a total of 45 lincRNAs was altered in *Pou4f2*-null retinas (18 up-regulated and 27 down-regulated) and 25 in *Isl1*-null retinas (12 up-regulated and 13 down-regulated). Among these differentially expressed lincRNAs, only three were upregulated and nine were downregulated in both *Pou4f2*-null and *Isl1*-null retinas.

## Discussion

A growing number of lncRNAs have been characterized for their biological roles in diverse cellular and molecular processes, including visual maintenance and impairment [7]. Recent omics strategy based studies have greatly increased our knowledge about lncRNAs in mouse eye / retina, such as their tissue specificity in mouse eye and their expression in particular retinal neuron cell [21, 23]. Nevertheless, so far there is still lack of a comprehensive annotation of lncRNAs expressed in all the cell types of mouse retina during its formation.

In this work, by using long-reads sequencing, we identified about 28,000 noncoding transcripts that were expressed in embryonic and neonatal mouse retinas and characterized 940 multiple-exon encoded lincRNAs. About 13% of these lincRNAs were transcribed from previously annotated genes, as well as about 78% of them were novel isoforms. Notably, 42% of these identified lincRNAs were transcribed from the unannotated loci of photoreceptors (392/940). Therefore, apart from providing a comprehensive data set of mouse lncRNAs, this work also indicates that, maybe ascribed to the high spatio-temporal specificity of lncRNA expression and the resolution of the short reads in distinguishing the isoforms, the current annotation of mouse lncRNAs is far from complete.

According to the study on lncRNAs expressed in 15 mouse tissues (Zhao et al., 2016), lncRNA genes in each tissue are closely related to the tissue’s physiological functions. By using microarray experiments, Chen et al. (2017) investigated the lncRNA genes expressed in different tissues of newborn (P0) and adult (8 week-old) mouse eyes and demonstrated the high functional correlation between the lncRNAs and the tissues. For instance, GO enrichment analysis of protein-coding genes adjacent to retina-specific lncRNAs showed that, similar to the observation of this work, several retinal cell specificity and visual function associated biological processes were overrepresented, such as retinal cone cell development, synapse assembly, axonogenesis and retina development in camera-type eye. In this work, a further investigation of lncRNA function was performed, by identifying the lincRNAs that were associated with the key RGC regulators. A total of 90 differentially expressed lincRNAs were identified after function loss of *Math5*, *Isl1* and *Pou4f2*, with more than a half of them (50/90) were not detected in the photoreceptors, indicating the close relation of these lincRNAs to the cellular processes in RGCs and the cell type specificity for some of them such as *linc-3a*, *−3b* and *-3c*, which were further supported by the FISH detection. We have also noticed that, despite the functional redundancy, *Pou4f2* and *Isl1* regulated a set of varied lincRNAs, as shown previously that they regulated distinct but overlapping set of protein-coding genes [51], suggesting the roles of lincRNAs in the precise regulatory networks of retinal neuron cell differentiation and development.

Consistent with the understanding that lncRNAs are commonly lowly expressed, most lincRNAs identified in this work showed a low abundance. However, we also found high abundance for some lincRNAs with several of them were widely expressed and functional associated with retinal development, i.e., *Miat*, *Malat1*, *Rncr3 and potentially Lockd*, raising up the possibility that the other high expressed lincRNAs could be involved in the regulation of retinal development as well. Similarly, further studies are needed to elucidate the importance in retina/eye development of many lincRNAs that are found nearby a series of key regulators of retinal cell differentiation and axon genesis.

Taking advantage of the long reads, we have identified thousands of noncoding transcripts in this work, demonstrating the high complexity of lncRNA expression in mouse retinas. Due to the fact that the DNA strand encoding these transcripts is unknown, their coding potentials were calculated for both strands, which sometimes leads to an increase of false positives for lncRNA prediction. In addition, the identified noncoding transcripts may also contain the ones overlapping protein-coding genes and potential fragments of alternative splicing forms of coding genes. It is noted that the average length of 940 retinal expressed lincRNAs is much longer than the known ones (about two-fold), but it is similar to that of the consensus sequences from iso-seq (with the average length about 2900 nt), indicating that the longer sequence is more likely due to the Pacbio long-reads sequencing technology, but not the strategy of lincRNA identification. However, as limited number of lincRNAs share the same structures with known ones, we could not conclude if iso-seq has generated more full-length transcripts of lincRNAs, although it is the design. It is also interesting that the number of single-exon encoded noncoding transcripts identified in this work is much more than expected. Inferred from the result of short reads mapping, i.e., at least two short reads could be aligned to 93% of the single-exon transcripts, most of the identified single-exon noncoding transcripts were expressed in the retinas. However, whether they are uncharacterized fragments of known genes or independent lncRNA genes needs to be further validated.

## Conclusions

Here, we reported a systematic annotation and function analysis of lincRNAs expressed in developmental mouse retinas. Long-reads sequencing and subsequent analysis in this study identified hundreds of retinal lincRNAs, which showed the high complexity of lncRNA genes in transcription and the high specificity in retina tissue. Further analysis revealed that the function of these lincRNAs were closely related to the retinal development, such as a series of lincRNAs were associated with the key regulators of RGC differentiation (i.e., *Math5*, *Isl1* and *Pou4f2*), including three lincRNAs enriched in RGCs. Summarily, this study provided a comprehensive annotation of mouse retinal lincRNAs and implied their crucial roles in the regulation of neuron cell differentiation and retinal development.

## Additional files

Additional file 1: **Table S1.** Statistics of reads from Iso-seq before and after short reads correction. (XLSX 13 kb)

Additional file 2: **Table S2.** PCR primer pairs used for lincRNA validation. Each primer pair spans at least one intron. (XLSX 14 kb)

Additional file 3: **Table S3.** Comparison of the chromosomal location between lincRNAs identified in this work and transcripts in mouse photoreceptors. Annotation of photoreceptor transcripts is public available in NCBI (Gene Expression Omnibus, GSE74660). (XLSX 51 kb)

Additional file 4: **Table S4**. Functional enrichment analysis of genes nearby potential retina-specific lincRNAs. (XLSX 19 kb)

Additional file 5: **Table S5.** Functional enrichment analysis of genes nearby lincRNAs that are differentially expressed after function loss of *Math5*, *Isl1* and *Pou4f2*. (XLSX 21 kb)

Additional file 6: **Figure S1.** Reverse transcription PCR amplification of ten randomly selected lincRNAs. Primer pairs are designed to amplify the fragments spanning at least one intron. All products (> 100 bp) are sequenced and analyzed. Bands representing the target region are indicated by red arrows. (TIF 3682 kb)

Additional file 7: **Figure S2.** Gene structures of *linc-3a* (A), *linc-3c* (B) and their overlapped genes**.** Blue boxes and lines between them indicate exons and introns, respectively. All the gene features are drawn to scale. (TIF 56 kb)

Additional file 8: **File S1**. Probes for in situ *hybridization* analysis of *linc-3a*, *linc-3b* and *linc-3c*. (DOC 33 kb)

Additional file 9: **File S2**. Chromosomal location of lincRNAs identified in mouse retina. Gene names are assigned when transcripts overlap known lncRNA genes (GENCODE version M17). (GTF 391 kb)

## Data Availability

All the raw data generated in this study are available in Sequence Read Archive (SRA) of NCBI under the project PRJNA514424 (www.ncbi.nlm.nih.gov/sra/PRJNA514424). RNA-seq data of retinas from wild type, *Math5*-null, *Isl1*-null and *Pou4f2*-null mice at E14.5 were downloaded from NCBI Sequence Read Archive (SRA) under the accession number SRP037539.

## Abbreviations

CSS: circular consensus sequence
FLNC: full-length non chimera
lincRNA: intergenic long noncoding RNA
lncRNA: long noncoding RNA
ncRNA: non-coding RNA
NFL: non full-length
ORF: open reading frame
RGC: retinal ganglion cell
RPC: retinal progenitor cell
