# Supplementary figures and images for: Systematic identification of intergenic long-noncoding RNAs in mouse retinas using full-length isoform sequencing
Source: BMC Genomics. 2019 Jul 8;20:559. doi: 10.1186/s12864-019-5903-y (PMC6615288; doi:10.1186/s12864-019-5903-y)

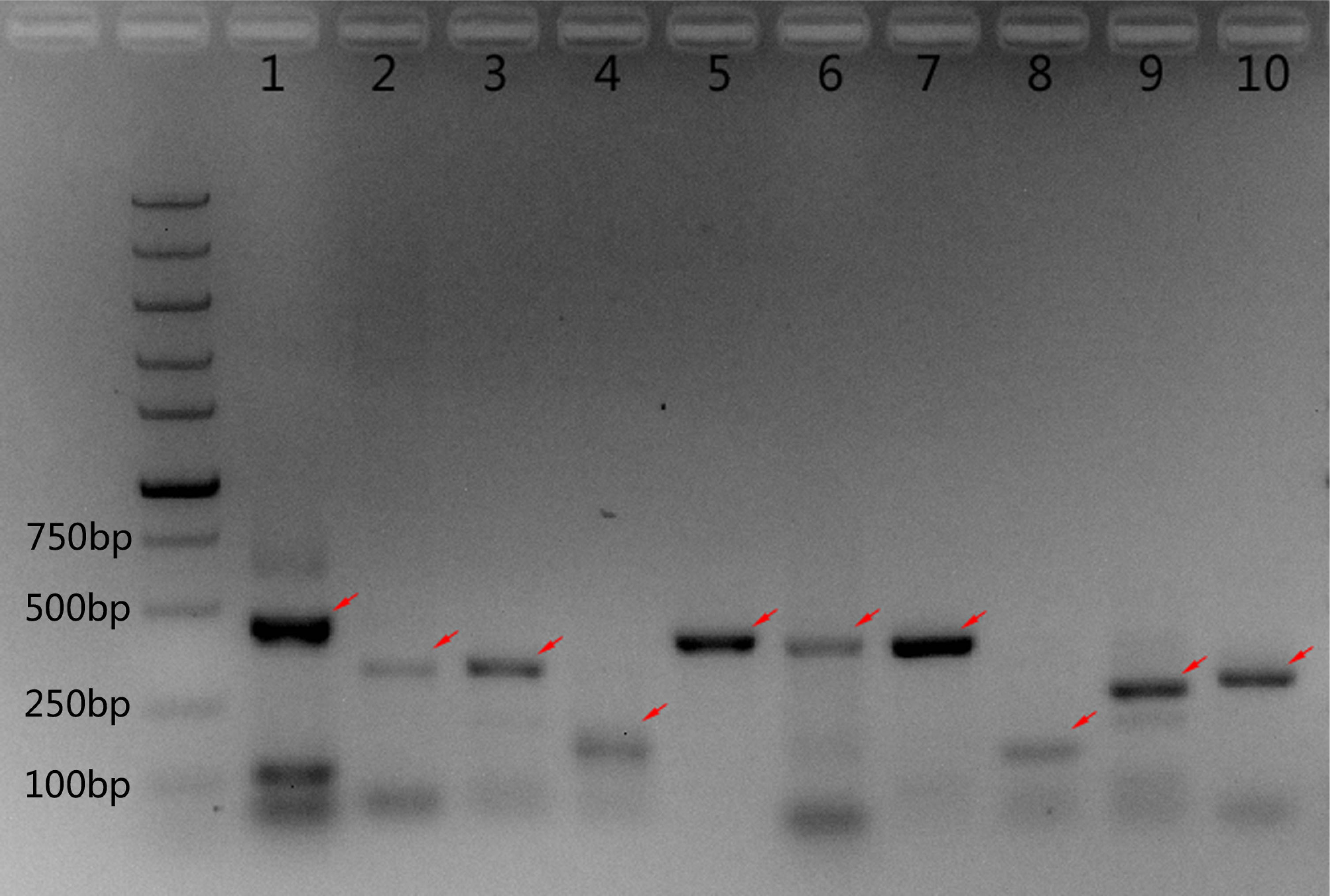

Supplement: Supplementary file 6 — Figure S1. Reverse transcription PCR amplification of ten randomly selected lincRNAs. Primer pairs are designed to amplify the fragments spanning at least one intron. All products (> 100 bp) are sequenced and analyzed. Bands representing the target region are indicated by red arrows. (TIF 3682 kb) [file 12864_2019_5903_MOESM6_ESM.tif]

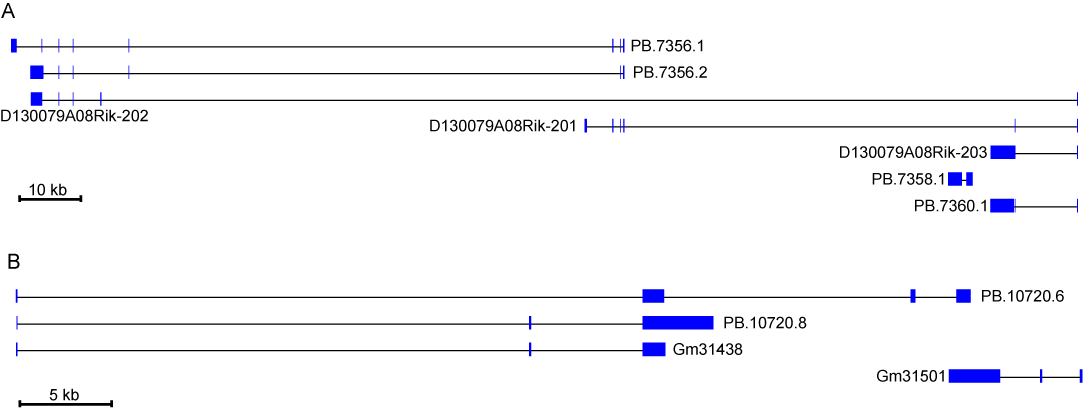

Supplement: Supplementary file 7 — Figure S2. Gene structures of linc-3a (A), linc-3c (B) and their overlapped genes. Blue boxes and lines between them indicate exons and introns, respectively. All the gene features are drawn to scale. (TIF 56 kb) [file 12864_2019_5903_MOESM7_ESM.tif]
