# Supplementary material for: Systematic identification of intergenic long-noncoding RNAs in mouse retinas using full-length isoform sequencing
Source: BMC Genomics. 2019 Jul 8;20:559. doi: 10.1186/s12864-019-5903-y (PMC6615288; doi:10.1186/s12864-019-5903-y)
Supplement: Supplementary file 8 — File S1. Probes for in situ hybridization analysis of linc-3a, linc-3b and linc-3c. (DOC 33 kb) [file 12864_2019_5903_MOESM8_ESM.doc]

>linc-3a (PB.7356.2) probe

T3-attaaccctcactaaagggaGAAAAGAATGCTGCATCTTTGGGACTTCGCTCCTAGTTGATAAGGATGAAACCATTTCAGAATGCCTGTGGTGTGTGCTCATGAGCAATGCCGAGACCGGAAGCTTCTAGTAGGCCTGAGGCACATTGAGCTCGCATCTTTGGTACAATGACTGACACAAGTTACAGTAGAAGAAAGAACTATAGACTATCATTCATTGGCCATGGACCTCCATCATTGAAGAAGCCATCTAAGATCCCATGTACTGTAAAGGAAGCCTTGAGAGGCATAGCTAAGATTGGATGTAATGGCATGTGGAACAGTTTAAGCAGTCAATGACCTTTTATTTTTCTTTCAACTCCAATATAGTCAGCAGAGTATAAACAAGATGAGAATTCAGCAGAACACATGTATTATAATTccctatagtgagtcgtatta-T7

>linc-3b (PB.21631.1) probe

T7-taatacgactcactatagACACTAGGTGCATATACCGATGAGGACCTGCGGGTGGCTGGTCCGGGACAGGGGTGATCCGGAACAGGTGCAGGCCCTGGGAGCTTGGGAGGGCCAGCAGGTGTGAGAGCAAGCAGAAGGCCTAAGAGGCACTGGCGCGAGCTAGGACAAAAACAAAGTGGAGGGCAAGCGTGGAAAGGGGAGCCCTCTGGGAGTCAGCACCCGGAAGCCCTGCGGCGGTAGCGGCGAACAGGAAACCGTGGATCCTCCCCTGACCTCATCCAGGCCGGGGCTGGCAGGTTCAAGTTAGGAAAGCTGTCGTCGGAATACTCCCCCCCCCCCCCCACCCCAAAGCCTCTGCTTTGACAACGCGATGGGATCTGTCCGTGGTGCTTAAATCTCTGCTGCTTTGCCAGTCTGGAGCCCGCCTTTCTCTTCCCCTGCGTAAAGGAAAATAATAAACAGTAGCCAAAGGGGATTCCTACGAGCCATGTTCTCCACTGAGAGCCCTAAGAAGCCTGGGCAAGGACTCGGAGAGGGGGGTGTTGACATGAAAGAACGTGGCTGTGTGCAAGACTGTTCAAAGAGACGCATGGGACTGCCCCCGGAGCTCTGTTGATGTTCCAGGCACTGGGAGGAATCAAAGCCACCTGGCAGAAAGCAAACTTCACTGCCTCTTCCCAGAGATAAAGGGAAAACTTACAAAAGGAGAGATTTGCAGACATCCAACAGGACTTGAATAAAGTCATGACTGCGACATCTGCTGGGAAGCCACCAGGAAGTGGAGGACTACAGCCCACGTCAAATCTCATGCATGTAGAGGATGTGAAGTCTCCTTCCTTCCTTCCCCATCCCCAGATGGCTTAGAAACAAAACAGCATACATTTTGTAGGAAACAGAAAACACCTAAGAAGAAATGCTAATGGGATGGCTGCAAAGATTATtccctttagtagggttaat-T3

>linc-3c (PB.10720.6) probe

T3-attaaccctcactaaagggaAAGGTCCTGTTGAGTTGCCCACAGTTGTGCCGTGAGCTCCAGGTCTCCAGGTCTTGCAAGCTGTCAACCATGATGGGGTAGCCTTTGGTCATACAGCTCTCTTGGTGCAGATGCCATGACTGCTGCCTGCAGAACTCATCAGTTACCGAAGAGACCTCCAAAGAAAAGAATGTGAATGAAGAATGAAGAATCCAGTGGAAGACAGACAGGTCCTTCAGCGTGGGGAGGACCTCAGCTACCATGTCTGGGGTAGCATTTTGTTCCCTGTCTGGTGTTGGAGGCTGATGGAGCTGCTCTGGCAAAGGCAGAAGGTGGTGGGCAGGGCTGGTGCTTTAACTCCTAACCCCGCTTTTATTGATGAGAGGCTCCCTGGACTTTCTGGGTTTCTTTCATCTGTAAAGCGAGAGGCCGAAGACACTGTTTTCTCTGACGTGAGTCTGTACACAAGGCAGTGCGCAGTCGGCAAATGAccctatagtgagtcgtatta-T7
